# CircFBXW7 inhibits the tumorigenesis of T-cell acute lymphoblastic leukemia through modulating miR-494-3p/SOX1 axis

**DOI:** 10.1038/s41420-022-00857-1

**Published:** 2022-05-10

**Authors:** Cong Luo, Jun-Jun Li, Feng Wen, Yi-Xiong Cao, Ze-Yu Luo, Xing-Xing Long

**Affiliations:** Department of Hematology, the First Affiliated Hospital, Hengyang Medical school, University of South China, Hengyang421001, Hengyang, Hunan Province China

**Keywords:** Cell biology, Haematological diseases

## Abstract

T-cell acute lymphoblastic leukemia (T-ALL) is a type of leukemia with high malignant behaviors, which seriously threatens the health of people. It has been reported that circFBXW7 is downregulated in lymphoblastic leukemia. Nevertheless, the exact role of circFBXW7 in T-ALL remains elusive. MTT assay was used to assess the cell viability. Cell apoptosis was assessed by flow cytometry. In addition, mRNA expressions were assessed by RT-qPCR, and a western blot was applied to investigate the protein levels. Meanwhile, the correlation among circFBXW7, miR-494-3p, and SOX1 was explored by RNA pull-down and dual-luciferase reporter assays. Furthermore, a xenograft mice model was conducted to verify the function of circFBXW7 in T-ALL in vivo. CircFBXW7 was significantly downregulated in T-ALL, of which overexpression inhibited the cell viability and induced the apoptosis of Jurkat cells. Moreover, miR-494-3p was identified to be a functional downstream effector to be involved in circFBXW7-mediated T-ALL cell proliferation. Besides, SOX1 was a direct target of miR-494-3p, and the impact of miR-494-3p mimics on T-ALL cell growth was inhibited in the presence of SOX1 overexpression. Furthermore, overexpression of circFBXW7 dramatically inhibited T-ALL tumor growth. In summary, circFBXW7 attenuated the tumorigenesis of T-ALL through the mediation of the miR-494-3p/SOX1 axis, which might be novel targets for T-ALL treatment.

## Introduction

Acute lymphoblastic leukemia (ALL) is known to be an aggressive tumor, which is correlated with a survival rate of 85% [1, 2]. In addition, ALL can be classified into two types: T-ALL and B-ALL according to the previous outcomes [3]. Meanwhile, T-ALL is derived from T cells which often accumulate genomic alterations, thus inducing the transformation [4]. At present, the recommended treatment for ALL is majorly a combination of anthracyclines, vincristine, and steroids [5, 6]. However, the survival rates within 5 years remain still not ideal [7]. Based on these backgrounds, it is essential to explore new methods for T-ALL treatment.

Circular RNAs (CircRNAs) are a type of endogenous RNAs with a stable structure [8]. In addition, circRNAs can act as crucial mediators in cell functions such as gene expression, protein synthesis, and modification of post-transcription [9, 10]. In addition, dysregulation of circRNAs was closely associated with the tumorigenesis of T-ALL. For example, Hou Y et al. found that circRNA_0000094 could restrain the T-ALL progression through modulation of miR-223-3p/F-box axis [11]; Buratin A et al. showed that circSTAM could modulate T-ALL cell proliferation [2]. Meanwhile, a previous study indicated that circFBXW7 was downregulated in pediatric acute lymphoblastic leukemia [12]. Nevertheless, the function of circFBXW7 in T-ALL remains largely unknown.

MicroRNAs (miRNAs) are noncoding small RNAs, which can mediate mRNA expression by mRNA translation inhibition [13]. In addition, it has been reported that miRNAs are crucial modulators for various diseases, including T-ALL. For instance, Xu K et al. revealed that miR-96-5p upregulation could inhibit the proliferation of T-ALL cells through targeting HBEGF [14]; Saccomani V et al. showed that miR-22-3p upregulation could attenuate the progression of T-ALL via regulation of Notch1 signaling [4]. Thus, miRNAs might act as key mediators in T-ALL. Meanwhile, it has been revealed that circRNAs could mediate the tumorigenesis of T-ALL via sponging miRNAs [2]. However, the correlation between circFBXW7 and miR-494-3p in T-ALL remains largely unknown.

In this study, we aimed to investigate the function of circFBXW7 in T-ALL. In addition, we sought to explore the mechanism by which circFBXW7 mediates the progression of T-ALL. We hope this research would shed new lights on exploring the new methods for T-ALL treatment.

## Results

### Overexpression of circFBXW7 inhibited the proliferation of Jurkat cells by promoting the apoptosis

**Fig. 1:**
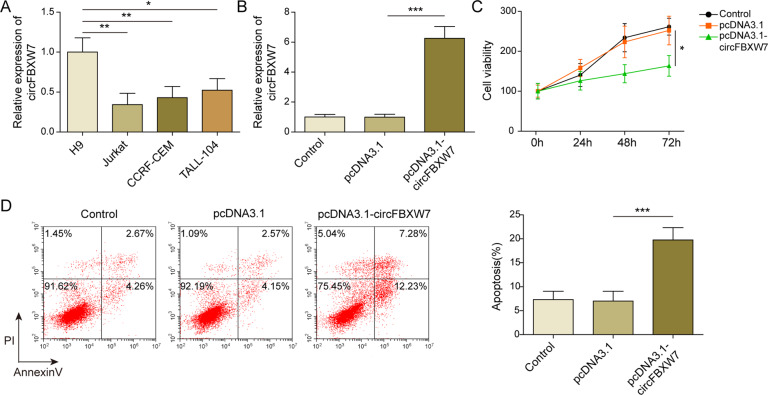
Overexpression of circFBXW7 inhibited the proliferation of Jurkat cells via inducing apoptosis. **A** RT-qPCR analysis showed that the expression of circFBXW7 in T-ALL cells was significantly lower, compared to that in H9 cells. **B** RT-qPCR analysis revealed that pcDNA3.1 or pcDNA3.1-circFBXW7 was stably transfected into Jurkat cells. **C**, **D** MTT and flow cytometry determination showed that overexpression of circFBXW7 inhibited the proliferation and induced the apoptosis of Jurkat cells. The quantitative values are expressed as a mean ± SD from three independent experiments. *P < 0.05, **P < 0.01, ***P < 0.001.

To investigate the role of circFBXW7 in T-ALL, RT-qPCR was performed. As indicated in Fig. 1A, the expression of circFBXW7 in T-ALL cells was significantly downregulated, compared to that in H9 cells. Since Jurkat cells were more susceptible to circFBXW7 expression, Jurkat cells were selected ifor subsequent analysis. In addition, the level of circFBXW7 in Jurkat cells was significantly upregulated by pcDNA3.1-circFBXW7 (Fig. 1B). Overexpression of circFBXW7 notably inhibited the viability of T-ALL cells (Fig. 1C). Moreover, enhanced circFBXW7 notably induced apoptosis in Jurkat cells (Fig. 1D). Taken together, overexpression of circFBXW7 inhibited the proliferation of Jurkat cells by promoting apoptosis.

### CircFBXW7 directly bound to miR-494-3p

**Fig. 2:**
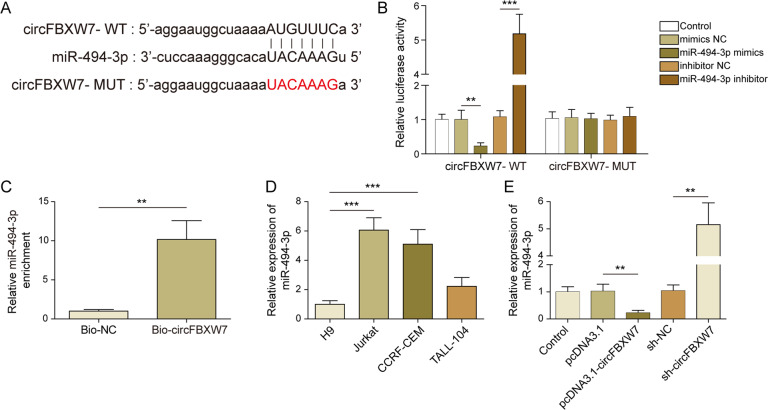
CircFBXW7 acted as a sponge of miR-494-3p. **A** Bioinformatic prediction showed that circFBXW7 had putative binding sites with miR-494-3p. **B** Dual-luciferase reporter assay indicated that circFBXW7 bound with miR-494-3p. **C** RNA pull-down suggested that miR-494-3p was enriched by ciircFBXW7. **D** RT-qPCR investigation showed that miR-494-3p was notably upregulated in T-ALL cells. **E** RT-qPCR detection revealed that miR-494-3p level was negatively regulated by circFBXW7. The quantitative values are expressed as a mean ± SD from three independent experiments. **P < 0.01, ***P < 0.001.

To find the downstream miRNA of circFBXW7, starbase was used. As shown in Fig. 2A, circFBXW7 had a putative binding site with miR-494-3p. Additionally, the luciferase activity in WT-circFBXW7 was decreased by miR-494-3p elevation (Fig. 2B). In contrast, miR-494-3p depletion markedly elevated the relative luciferase activity (Fig. 2B). Meanwhile, the enrichment of miR-494-3p was significantly increased by bio-circFBXW7 (Fig. 2C), and miR-494-3p was notably upregulated in T-ALL cells (Fig. 2D). Furthermore, the level of miR-494-3p in T-ALL cells was significantly downregulated by pcDNA3.1-circFBXW7, while circFBXW7 knockdown notably increased the expression of miR-494-3p (Fig. 2E). In summary, circFBXW7 directly bound to miR-494-3p.

### CircFBXW7 suppressed the growth of T-ALL cells through the mediation of miR-494-3p

**Fig. 3:**
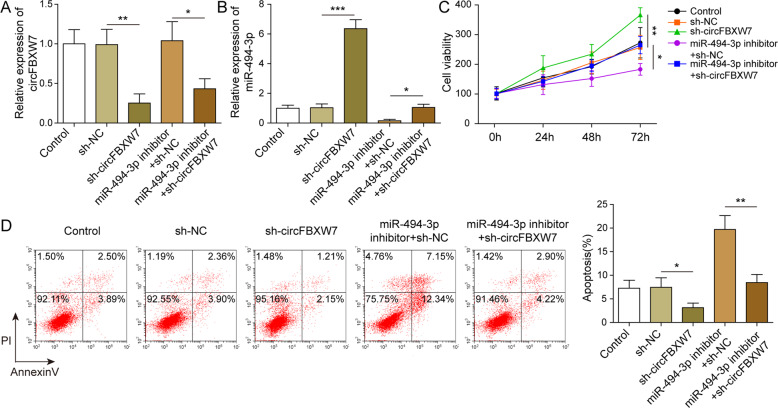
CircFBXW7 inhibited the growth of T-ALL cells through the mediation of miR-494-3p. **A, B** RT-qPCR detection manifested that miR-494-3p was dramatically increased in the presence of sh-circFBXW7. **C**, **D** MTT and flow cytometry determination denoted circFBXW7 inhibited the viability and induced the apoptosis of T-ALL cells through the mediation of miR-494-3p. The quantitative values are expressed as a mean ± SD from three independent experiments. *P < 0.05, **P < 0.01, ***P < 0.001.

For assessing the mechanism by which circFBXW7 mediates the growth of T-ALL cells, RT-qPCR was performed. The data indicated that the expression of circFBXW7 in T-ALL cells was significantly decreased and miR-494-3p was dramatically increased in the presence of sh-circFBXW7, while miR-494-3p inhibition did not affect the level of circFBXW7 (Fig. 3A). In addition, sh-circFBXW7 significantly increased the level of miR-494-3p in T-ALL cells, while the impact was abolished by miR-494-3p depletion (Fig. 3B). Moreover, sh-circFBXW7 significantly increased the viability of T-ALL cells, which was restored by miR-494-3p repression (Fig. 3C). Consistently, miR-494-3p inhibitor notably reversed the antiapoptotic effect of sh-circFBXW7 (Fig. 3D). To sum up, circFBXW7 inhibited the growth of T-ALL cells through the mediation of miR-494-3p.

### SOX1 was a direct target of miR-494-3p

**Fig. 4:**
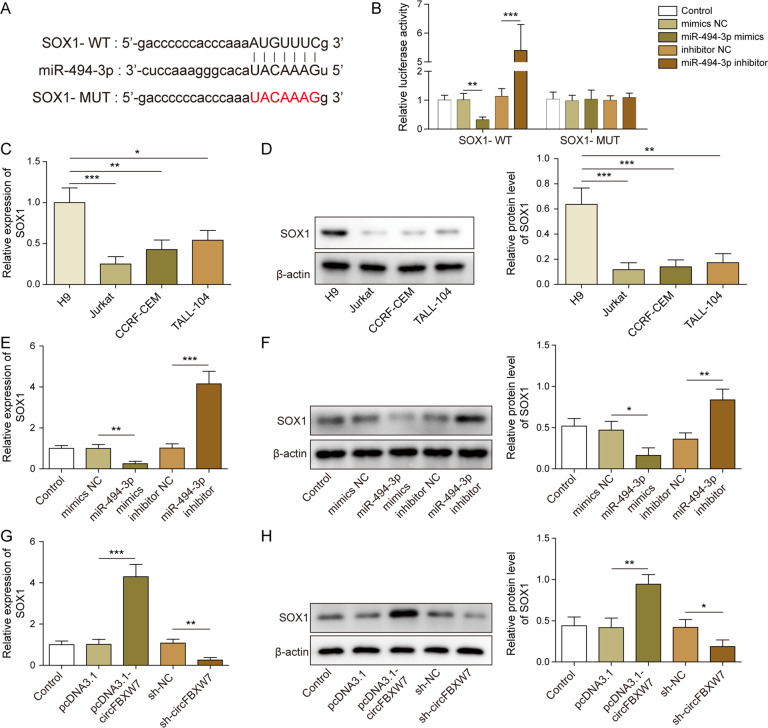
SOX1 was a target of miR-494-3p. **A** Targetscan prediction showed that SOX1 was targeted by miR-494-3p. **B** Dual-luciferase reporter assay detection revealed that miR-494-3p could bind with SOX1. **C**, **D** RT- qPCR and Western blot analysis indicated SOX1 level was lower in T-ALL cells. **E**, **F** RT-qPCR and Western blot analysis showed that SOX1 level was negatively regulated by miR-494-3p. **G**, **H** RT- qPCR Western blot detection indicated that SOX1 level was positively regulated by circFBXW7. The quantitative values are expressed as a mean ± SD from three independent experiments. *P < 0.05, **P < 0.01, ***P < 0.001.

In order to explore the downstream mRNA of miR-494-3p, targetscan was performed. As revealed in Fig. 4A, SOX1 was identified to be the downstream target of miR-494-3p, and miR-494-3p negatively modulated the luciferase level in WT-SOX1 (Fig. 4B). Meanwhile, the level of SOX1 in T-ALL cells was significantly downregulated in T-ALL cells, compared with that in H9 cells (Fig. 4C, D). In addition, miR-494-3p mimics notably inhibited the expression of SOX1 in T-ALL cells, while miR-494-3p inhibitor exhibited the opposite effect (Figs. 4E, F). Besides, SOX1 level in T-ALL cells was positively regulated by circFBXW7 (Fig. 4G, H). Taken together, SOX1 was found to be the direct target of miR-494-3p.

### miR-494-3p increased T-ALL cell proliferation by targeting SOX1

**Fig. 5:**
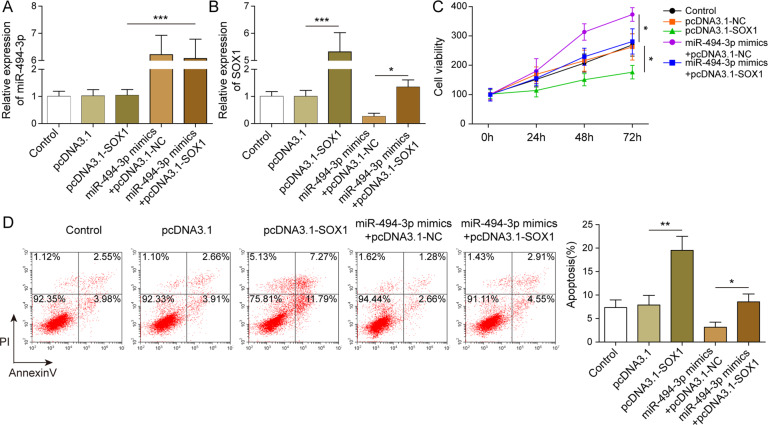
miR-494-3p induced T-ALL cell proliferation by targeting SOX1. **A**, **B** RT-qPCR detection showed that miR-494-3p targeted SOX1. **C**, **D** MTT and flow cytometry investigation revealed miR-494-3p promoted the viability and inhibited the apoptosis of T-ALL cells via targeting SOX1. The quantitative values are expressed as a mean ± SD from three independent experiments. *P < 0.05, **P < 0.01, ***P < 0.001.

To confirm the relation between SOX1 and miR-494-3p, RT-qPCR was performed. The data revealed that pcDNA3.1-SOX1 induced upregulation of SOX1 while it did not exert effects on the expression of miR-494-3p (Fig. 5A), suggesting that SOX1 might be a downstream target of miR-494-3p. Meanwhile, the level of SOX1 in T-ALL cells was notably increased in the presence of pcDNA3.1-SOX1, and miR-494-3p mimics-induced inactivation of SOX1 was obviously reversed in the presence of pcDNA-SOX1 (Fig. 5B). Moreover, overexpression of SOX1 could reverse miR-494-3p mimics-induced promotion of cell viability (Fig. 5C). Consistently, the antiapoptotic effect of miR-494-3p mimics was partially reversed in the presence of pcDNA3.1-SOX1 (Fig. 5D). To sum up, miR-494-3p promoted the proliferation of T-ALL cells via targeting SOX1.

### Overexpression of circFBXW7 significantly inhibited the tumor growth of T-ALL in vivo

**Fig. 6:**
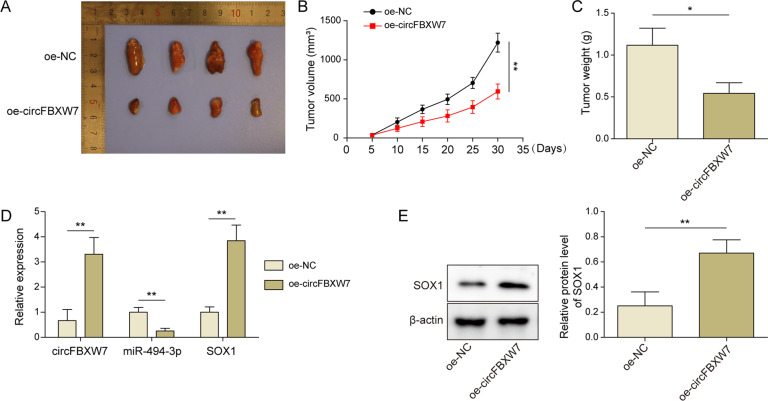
Overexpression of circFBXW7 significantly inhibited the tumor growth of T-ALL in vivo. **A** The tumor tissues of mice were collected and pictured. **B** CircFBXW7 overexpression inhibited the tumor size of mice. **C** CircFBXW7 overexpression inhibited the tumor weight of mice. **D** The levels of circFBXW7, miR-494-3p, and SOX1 in tissues of mice were detected by RT-qPCR. **E** Western blot detection showed that circFBXW7 positively regulated SOX1 level in tumor tissues of mice. The quantitative values are expressed as a mean ± SD from three independent experiments. *P < 0.05, **P < 0.01.

To further verify the function of circFBXW7 in T-ALL, a xenograft mice model was established. As shown in Fig. 6A, B, the tumor size of mice was significantly reduced in the presence of oe-circFBXW7. Consistently, the tumor weight of mice was notably decreased by circFBXW7 overexpression (Fig. 6C). Meanwhile, oe-circFBXW7 markedly increased the level of circFBXW7 and SOX1 and inhibited the expression of miR-494-3p in tissues of mice (Figs. 6D, E). In summary, overexpression of circFBXW7 significantly inhibited the tumor growth of T-ALL in vivo.

## Discussion

It has been reported that T-ALL is a hematological malignant tumor which resulted from oncogenic transformation [15]. In addition, T-ALL is related with limited clinical outcomes, while advances in intensified chemotherapy have increased the rates of pediatric and adult cures [11, 16]. In this work, we explored the detailed function of circFBXW7 in T-ALL. As expected, circFBXW7 was found to be downregulated in T-ALL cells, and circFBXW7 overexpression could inhibit the growth of T-ALL cells through the mediation of the miR-494-3p/SOX1 axis. Thus, our research might supply a new perspective on T-ALL treatment.

Our findings revealed that circFBXW7 could inhibit the proliferation and induced apoptosis in T-ALL cells, and suppressed the tumor growth of T-ALL. Previous studies have revealed that circFBXW7 could act as an inhibitor during cancer progression [17, 18]. In addition, a recent study indicated that circFBXW7 was downregulated in pediatric acute lymphoblastic leukemia [12]. Consistently, our research indicated that circFBXW7 upregulation could inhibit the tumorigenesis of T-ALL through sponging miR-494-3p, which further supplemented the underlying mechanism of circFBXW7 in T-ALL.

This research revealed that circFBXW7 could bind to miR-494-3p in T-ALL cells. miR-494-3p plays key role in cancer progression. For instance, miR-494-3p upregulation could promote the growth of NSCLC cells [19]; Wei W et al. found miR-494-3p could enhance methotrexate sensitivity in osteosarcoma cells [20]. Our data firstly found the function of miR-494-3p in T-ALL, suggesting that miR-494-3p could act as an oncogene in T-ALL. On the other hand, the correlation between circFBXW7 and miR-494-3p has never been reported before. Therefore, our study firstly found miR-494-3p could be bound to circFBXW7 in T-ALL cells. Meanwhile, Ye F et al. found miR-197-3p could be sponged by circFBXW7 in breast cancer cells [18]; Gao ZG et al. revealed that circFBXW7 could alleviate the progression of glioma via the mediation of the miR-23a-3p/PTEN axis [17]. Thus, more miRNAs sponged by circFBXW7 in T-ALL need to be explored in the future.

The transcription factor SOX1 is a member of the SOX family transcription factors with a high-mobility group domain [21]. In addition, SOX1 is involved in the progression of multiple cancers (cervical cancer, gastric cancer, et al) [22, 23]. This research found SOX1 is the target of miR-494-3p in T-ALL cells, and SOX1 overexpression could reverse miR-494-3p mimics-induced T-ALL cells growth. Thereby, our research firstly revealed the function of SOX1 in T-ALL, suggesting that SOX1 might be a suppressor in T-ALL.

Obviously, there are some shortcomings in this work: (1) more miRNAs involved in T-ALL tumorigenesis need to be further explored; (2) more downstream mRNAs of miR-494-3p are needed to be found. Therefore, more investigations are needed in the coming future.

In conclusion, circFBXW7 overexpression inhibited the tumorigenesis of T-ALL through the mediation of the miR-494-3p/SOX1 axis. Therefore, our findings would shed new lights on exploring new strategies for T-ALL treatment.

## Material and methods

### Cell culture

T-ALL cell lines (Jurkat, CCRF-CEM, and T-ALL-104), Human T lymphocyte H9 cell lines (a derivative of HuT 78), and HEK-293T cells were purchased from the Chinese Academy of Sciences (Shanghai, China). H9 cell lines were used as normal references in this study. All Cells were maintained in RPMI-1640 medium (Lonza, Basel, Switzerland) containing 10% FBS (Thermo Fisher Scientific) and penicillin (100 U/mL), 100 mg/ml streptomycin (Gibco, Thermo Fisher Scientific, Waltham, MA, USA), and 2mM l-glutamine in 5% CO_2_ at 37 °C. Cells growing in log phase at a density of 1.0 × 10^6^ cells/ml were used for experiments.

### Cell transfection

Jurkat cells were transiently transfected with pcDNA3.1 or pcDNA3.1-circFBXW7 by using Lipofectamine 2000 (Invitrogen, MA, USA) for 24 h according to the protocol of the manufacturer. pcDNA3.1 and pcDNA3.1-circFBXW7 were purchased from Beyotime (Shanghai, China). For miR-494-3p transfection, Jurkat cells were transfected with miR-494-3p mimics, miR-494-3p inhibitor, or their corresponding negative controls including NC mimics or NC inhibitor (GenePharma, Shanghai, China) by Lipofectamine 2000 (Invitrogen, MA, USA).

Lentiviral vectors expressing circFBXW7 (oe-circFBXW7) and shRNA-directed target circFBXW7 (sh-circFBXW7) and their corresponding negative controls (oe-NC, sh-NC) were bought from GenePharma. For transfection, HEK-293T cells were transfected by Lipofectamine 2000 (Invitrogen, MA, USA). After transfection, the cells were incubated at 37 ˚C, and then the supernatant was collected. Supernatants of oe-circFBXW7 or sh-circFBXW7 and negative control were filtered into particles. Lentiviral particles were used to infect the cells. Puromycin (2.5 μg/mL, Sigma Aldrich, St. Louis, MO, USA) was applied to select stable cells after incubation.

### MTT assay

Cell viability in different groups was tested by MTT assay (Beyotime, Shanghai, China) based on the manufacturer’s protocol. Jurkat cells (5 × 10^3^ per/well) were seeded in 96-well plates overnight in the condition of 37 °C and 5% CO_2_. After 24, 48, or 72 h of treatment, cells were treated with 10 μl MTT solutions (Beyotime, Shanghai, China) and further incubated for 2 h at 37 °C. The absorbance (450 nm) was assessed by a microplate reader (Thermo Fisher Scientific, MA, USA). MTT absorbance was assessed three times in independent experiments.

### Cell apoptosis

Jurkat cells were trypsinized, washed with phosphate-buffered saline, and resuspended in Annexin V Binding Buffer. Subsequently, cells were stained with 5 μl FITC and 5 μl propidium iodide (PI) in the dark for 15 min at room temperature and maintained in the dark. Then cells were analyzed by flow cytometry (FACSCanto™ II, BD Biosciences, CA, US) to analyze the cells, and the rate of cell apoptosis was measured.

### RNA pull-down

Probe-control or probe-circFBXW7 from sh-circFBXW7 vector was transcribed and labeled by Biotin RNA Labeling Mix (Roche, Basel, Switzerland). An RNA structure buffer (Thermo, MA, USA) was used to induce secondary structure formation from the biotin-labeled RNAs. The biotinylated circFBXW7 and negative control (bio-NC) were generated via GenePharma and coated to streptavidin-conjugated magnetic beads. Magnetic beads were applied to incubate the cells for 6 h after cells were lysed. The RNA on the beads was isolated and RT-qPCR was applied to assess the enrichment of miR-494-3p.

### Dual-luciferase reporter assay

The partial sequences of circFBXW7 and the SOX1 3′-UTR containing the sites of miR-494-3p were synthesized by GenePharma. The aforementioned sequences were cloned into the pmirGLO vectors (Promega, Madison, WI, USA) for the establishment of wild-type (WT) or mutant (WT) reporter circFBXW7 and SOX1 vectors. The circFBXW7/SOX1-WT or MUT vector was transfected into cells along with miR-494-3p mimics using Lipofectamine 2000 reagent (Invitrogen, MA, USA). The data were quantified and normalized to Renilla luciferase activity.

### Reverse transcription-quantitative polymerase chain reaction

Reverse transcription-quantitative polymerase chain reaction (RT-qPCR) was used to examine the expression levels of circFBXW7, miR-494-3p, and SOX1 in each experimental group. MiRNA was isolated using miRNeasy Mini Kit (Qiagen). TaqMan MicroRNA Assay (Applied Biosystems) was used to determine the levels of miR-494-3p, and RT-qPCR was performed on Applied Biosystem 7500. Total RNA was extracted from Jurkat cells or tissues was isolated using TRIzol reagent (Takara, Dalian, China), according to the manufacturer’s protocol. In order to quantify the amount of mRNA, miRNA and circRNA were converted into cDNA using PrimeScript RT-PCR Kit (Takara). The amplification protocol was set as below: Incubation at 95 °C for 5 min, then 40 cycles of 10 s at 95 °C and 30 s at 60 °C. 2^−ΔΔCt^ was used for quantification. The primer for circFBXW7, miR-494-3p, SOX1, U6, and β-actin were designed from GenePharma. The β-actin and U6 were used as controls to normalize the expression of mRNA and miRNA. The sequences for primers were listed in Table 1.

**Table 1:** The sequences for primers.

Gene	Sequence of primer
circFBXW7	Forward: 5′-TCTCACCTTTCAAACGCTTGTC-3′
	Reverse: 5′-ACTCTCCTTCCTTTCCTCTGATTC-3′
SOX1	Forward: 5′-GGACTATGTATTGGTCCCTACCG-3′
	Reverse: 5′-TCGATGGTTGCAATGGTGTC-3′
miR-494-3p	Forward: 5′-AGGGAGGTGTCATCTCAACTGA-3′
	Reverse: 5′-CTCAACTGGTGTCGTGGAGTC-3′
U6	Forward: 5′-CTCGCTTCGGCAGCACAT-3′
	Reverse: 5′-AACGCTTCACGAATTTGCGT-3′
β-actin	Forward: 5′-GTCCACCGCAAATGCTTCTA-3′
	Reverse: 5′-TGCTGTCACCTTCACCGTTC-3′

### Western blot

RIPA (Beyotime, Shanghai, China) was applied to isolate total protein. BCA (Thermo Fisher Scientific) was used to quantify the total protein. SDS-PAGE (10%) was used to separate proteins (40 μg per lane), and then proteins were transferred onto PVDF membranes (Thermo Fisher Scientific). The membranes were incubated with primary antibodies against SOX1 (1:1000, #ab109290, Abcam, Cambridge, UK) and β-actin (1:5000, #ab8226, Abcam, Cambridge, UK) at 4 °C overnight after being blocked with 5% skimmed milk for 1 h. After that, the membranes were incubated with secondary antibodies HRP-conjugated (1:5000, #ab20272, Abcam, Cambridge, UK) for 1 h at room temperature. Protein bands were visualized using the enhanced chemiluminescent (ECL) kit (Thermo Fisher Scientific). β-actin was used for normalization. Image-Pro Plus 6.0 was applied for densitometric analysis.

### In vivo study

BALB/c nude mice (*n* = 8 per group; 6–8 weeks old) were bought from Vital River (Beijing, China). The mice were housed within a dedicated SPF facility. The oe-circFBXW7 was stably expressed in Jurkat cells (3 × 10^6^ cells, 200 μl volume) were transplanted subcutaneously in each mouse. Tumor volumes were calculated by the standard formula of Tumor size = (Length × Width × Width)/2 for every 5 days. At the end of the experiments, mice were euthanized and the tumors were collected and weighted. Tumor weights were measured using a balance. Afterward, four representative tumors were shown. All in vivo experiments were performed in accordance with the National Institutes of Health guide for the care and use of laboratory animals, following a protocol approved by the Ethics Committees of the First Affiliated Hospital, Hengyang Medical College, University of South China..

### Statistical analysis

All data were expressed as mean ± standard deviation (SD). Differences were compared using Student’s *t*-test (two groups) or one-way ANOVA analysis and Tukey’s tests (multiple groups). *P* < 0.05 was considered as significant changes.

## Data Availability

The datasets used or analyzed during the current study are available from the corresponding author on reasonable request.
